# Supplementary material for: Mucosal immunity of mannose-modified chitosan microspheres loaded with the nontyepable Haemophilus influenzae outer membrane protein P6 in BALB/c mice
Source: PLoS One. 2022 Jun 10;17(6):e0269153. doi: 10.1371/journal.pone.0269153 (PMC9187061; doi:10.1371/journal.pone.0269153)
Supplement: S1 Raw images — (DOCX) [file pone.0269153.s001.docx]

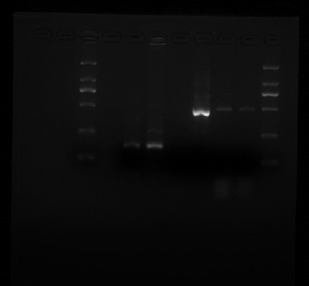

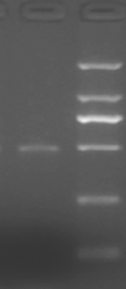

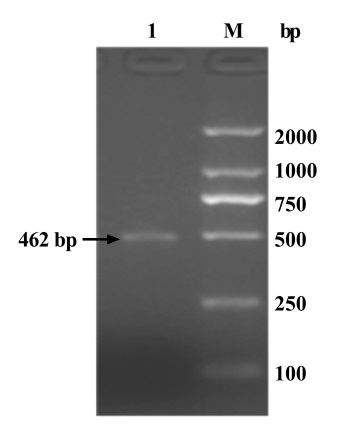


Original figure of Amplification product for the NTHi-P6 gene by PCR


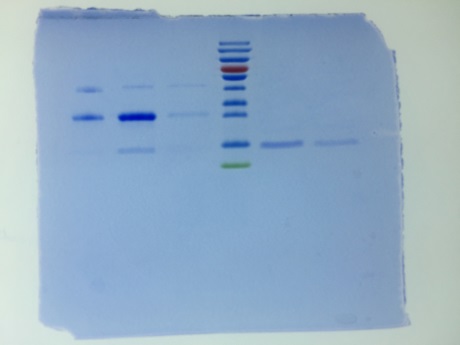

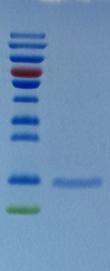

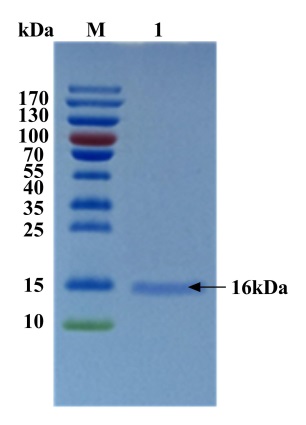


Original figure of SDS-PAGE gel analysis of tag-removed P6 protein expressed from the NTHi-P6 gene





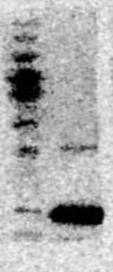

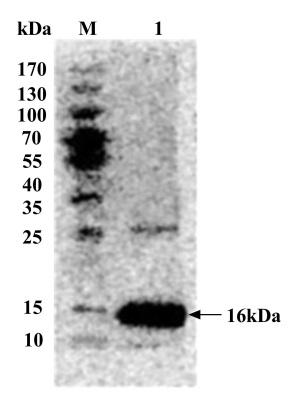


Original figure of Western blot analysis of tag-removed P6

**Fig. 2. Gene Cloning and Expression of the Loaded Antigen P6.**

Fig. 2. Gene Cloning and Expression of the Loaded Antigen P6.
