# Supplementary material for: Mucosal immunity of mannose-modified chitosan microspheres loaded with the nontyepable Haemophilus influenzae outer membrane protein P6 in BALB/c mice
Source: PLoS One. 2022 Jun 10;17(6):e0269153. doi: 10.1371/journal.pone.0269153 (PMC9187061; doi:10.1371/journal.pone.0269153)
Supplement: Attachment — Submitted filename: Response to Reviewers.docx [file pone.0269153.s002.docx]

Reviewer #1:

1 The concerns regarding the methodologies used, the sub-optimal description of methodologies, group sizes.?

Answers: The date of study groups with control group were analyzed utilizing an unpaired T test, such as P6 with PBS, P6-CMs with PBS, P6-MCMs with PBS, P6-MCMs with MCMs. While the compare among study groups P6, P6-CMs, P6-MCMs were analyzed by ANOVA test. 95 mice total were contributed for this study, 15 mice every group, 15 mice candidate. The available date of 3 mice every group were analysed statistically for antibody, cytokines, lymphocyte proliferation and T lymphocyte subpopulation assay. 4 mice every group for histopathologic examination.

2. The questions regarding the short time interval between immunization and challenge, and whether any immune response analyses were conducted after challenge.?

Answers: Immunization and challenge time were based on our Laboratory animal protocol, but we will try to extend immunization and challenge time according to your kindly suggestion. Furthermore , I’m so sorry that I have no time to conduct any immune response analyses after challenge to prove the effectiveness of the vaccine from multiple angles because of the graduation date, so that this should be a limitation of the study.

3. Additional information on the animal research and ensure you have included details on (1) methods of sacrifice, (2) methods of anesthesia and/or analgesia, and (3) efforts to alleviate suffering.?

Answers: Mice were anesthetized via intraperitoneal injection of sodium pentobarbital (1%, 50 mg/kg) and sacrificed via rapid dislocation of the necks. All efforts were made to minimize animal suffering and to reduce the number of animals used.

4. Only female mice were utilized, was the impact of gender on immune responsiveness to the vaccine considered. This should be cited as a limitation of the study.?

Answers: In this study, only female mice were used, merely considering that female mice usually live in peace, they are easier to raise in groups than male mice.

5. How were T cells isolated beyond the use of LSM? If only LSM was used how can you be sure that the response that was evaluated was a T cell response and not a result of other contaminant cells.?

Answers: The cytokines in splenic lymphocytes were measured, lymphocytes were isolated from spleen tissue with lymphocyte separation medium. The same as the splenic lymphocyte proliferation assay.

6. A complete description briefly describing the separation protocol and how lavage and blood samples were obtained should be included or at a minimum referenced. How were the mice sacrificed for histopathology.?

Answers: Blood samples were collected via the retro-orbital sinuses into drop tubes. The collection methods of lavage are as follows: The mice were sacrificed via rapid dislocation of the necks and dissected subsequently, the trachea was exposed and ligated from the middle section. After then, 500 μl PBS was injected in the trachea toward the lungs, thus the lungs lavage were obtained from the trachea after 5 minutes with gently kneading lung. Injecting 500 μl PBS toward the nasopharynx the same way, collecting the fluid flowing out of the nasal cavity, thus the nasal cavity lavage were obtained after repeating three times.

7. State in the methods when following vaccination, the mice were inoculated with NTHi.?

Answers: Mice vaccination and inoculation with NTHi were all performed via intranasal drip. the mice were anesthetized and challenged with NTHi (ATCC 49247) in a bacterial suspension containing an dose (LD100) (1×108 CFU/ml) via intranasal drip via intranasal drip after the last immunization

8. Which data were analyzed utilizing an unpaired T test.?

Answers: The date of study groups and control group were analyzed utilizing an unpaired T test, such as P6 and PBS, P6-CMs and PBS, P6-MCMs and PBS, P6-MCMs andMCMs

9. Paragraph starting on line 300 should be reworded based on response to the above described concerns with the methods. Further the use of the word “efficient” seems like an overstatement Based on the appearance of the graph the biological significance of this result would be questionable.

Answers: From the experimental data, the use of the word “efficient” seems like an overstatement, in addition, anyother immune response analyses were not conducted after challenge with bacteria, so that there lack of sufficient evidence to confirm the “efficient” of vaccine. We reword the word “efficient” as “effective”.

9. Did a pathologist review the tissue slides or who provided the changes described in the manuscript? What is meant by the worded “tended” to be normal? How many mice were utilized for this experiment? At what magnification were these images from?

Answers: We have consulted the pathologist in North University of Hebei for the histopathological changes. “tended to be normal” reworded “the nasal mucosa and lung tissues had a mild influx of inflammatory cells”. 4 mice every group for histopathologic examination, and 40 times magnification for these images.

10. Sentence starting on line 346 is overstated as antigen uptake and presentation were not directly assessed in these experiments. Further these results did not t show that P6-CM or P6-MCM “prevented” infection as infection was not assessed. Perhaps you are referring to evidence of disease based on histopathologic evaluation?

Answers: I’m so sorry the I have no time to conduct any immune response analyses after challenge to prove the effectiveness of the vaccine against NTHi from multiple angles. The sentences of antigen uptake and presentation were removed and reworded accordingly. But it showed that the microsphere vaccine can really reduce the invasion of NTHi to lung tissue and nasal mucosa tissue.

11. What is meant by “deploy” the mucosa (line 411)?

Answers: It reworded as “set up biological barriers on the surface of mucosa.”

12. Sentence starting on line 416 is overstated as colonization or possible invasion were not evaluated herein.

Answers: It removed the sentence “to prevent NTHi from invading”. And reworded “eliminate the invaded NTHi” as “weaken the invasion of NTHi”

13. Sentence staring on line 423 based on figure 5 I disagree that P6-CM “only” induces a Th2 response. Maybe the magnitude of production was greatest for IL-4, P6-CM did in fact induce production of the other cytokines to varying degrees. What were the p-values for the other cytokines for P6-CM vs. controls? The immune response to most antigens is a mixed response but in some cases one type of response predominates which I agree may be the case for P6-CM. Consider re-wording.?

Answers: It reworded as “the chitosan microsphere vaccine maybe mainly stimulate a Th2-type cellular immune response”. We also add the p-values for the other cytokines for P6-CM vs. controls on figure 5.

Reviewer #2:

1. Authors didn’t mentioned how many mice were used in each group?

Answers: 95 mice total were contributed for this study, 15 mice every group, 15 mice candidate. The available date of 3 mice every group were analysed statistically for antibody, cytokines, lymphocyte proliferation and T lymphocyte subpopulation assay. 4 mice every group for histopathologic examination.

2. All the studies were conducted two weeks after last vaccination. Were these studies done at later time points?

Answers: All the studies were conducted two weeks after last vaccination and done at later time points.

3. In the Figure 4 where IgA levels were measured in the Nasal and lung lavage fluids, were the samples pooled or measured individually?

Answers: IgA levels were measured in the Nasal and lung lavage fluids individually.

5. After final vaccination mice were challenged with nontypable Haemophilus influenzae tissues were collected and histopathologic examination was done. Were any other studies were done besides histopathologic examination?

Answers: I’m so sorry that I have no time to conduct any immune response analyses after challenge to prove the effectiveness of the vaccine from multiple angles because of the graduation date, so that this should be a limitation of this study.
