# Supplementary material for: Mucosal immunity of mannose-modified chitosan microspheres loaded with the nontyepable Haemophilus influenzae outer membrane protein P6 in BALB/c mice
Source: PLoS One. 2022 Jun 10;17(6):e0269153. doi: 10.1371/journal.pone.0269153 (PMC9187061; doi:10.1371/journal.pone.0269153)
Supplement: Attachment — Submitted filename: Response to Reviewers.docx [file pone.0269153.s003.docx]

Reviewer #2:

1. Did the authors compared bacterial burden between the groups after infection?

Answers: When the experiment was originally designed, it did include the bacterial burden in the mouse nasal cavity, but later due to the difficultly available NTHI in the nasal cavity and various operational reasons, the bacterial load experiment failed to proceed as scheduled.

2. Were Cytokine Levels in Spleen Lymphocyte Culture Supernatants and T Lymphocyte Proliferation Assays compared between groups after infection?

Answers: We only measure the cytokines in the spleen lymphocyte culture supernatants, while not compare the cytokine levels in the splenic lymphocyte culture supernatants and the T lymphocyte proliferation assays between the groups. We will take your suggestions seriously and design experiments in the future. It can further confirm the changes in cytokine secretion after cell proliferation.

3. Is there any pathological scoring of tissues performed??

Answers: When the results of the experiment came out, I consulted a professional pathologist to observe the obvious changes in inflammatory cell infiltration between the groups, especially in the degree of congestion and destruction of the lung tissue network structure, and cilia lodging and defect in the nasal mucosa group. These obvious changes can be observed with the naked eye, so we don’t have performed pathological scoring of tissues

Reviewer #3:

1. As a proof principle, the study lacks details on generation of microspheres. The authors argue that mannose modification of chitosan helps in better uptake of the microsphere by the immune cells. Since, the modification is “homemade” at about 19% efficiency, it is not clear if the desired uptake is achieved or not. Do the authors enrich modified chitosan before microspheres generation? Did the authors check if the cellular uptake of modified chitosan was increased in vitro? Do they achieve similar degree of immune response if a commercial (modified) version is used (if available)?

Answers: The detailed methods have been presented in lines 140-150, including the ratio of chitosan and TPP, PH of solutions, and the reaction time. Microspheres were prepared by the ionotropic gelation process following the report of Jiang et al and optimized. If the degree of substitution is too large, it is not conducive to the formation of spheres. Studies have found that when the degree of substitution of free amino groups in chitosan is 5% to 30%, the formation of microspheres will not be affected. It is stated that the method of loading P6 antigen with mannose-modified chitosan microspheres can enhance the P6 immune response. As for the degree of substitution of mannose modification that can maximize the efficiency of the mannose receptor method, we have not studied too much. We did not carry out the process of mannose targeting the mannose receptor in vitro, which is also a defect of this article, but it can infer that MCMs was captured through receptor-mediated endocytosis by targeting MR, which improves the antigen uptake efficiency of APC according the experimental data, so that enhanceing the efficiency of antigen presentation.

2. How consistent is the dimension of the microspheres formed? It is not clear how many times the microspheres were consistently generated and if each time it elicited similar degree of immune response.?

Answers: The uniformity of the formed microspheres was good, and the distribution of the microspheres was measured. It shows the distribution of the diameter of the microspheres by intensity in Fig 3. The formation method of the microspheres is the reference Microspheres were prepared by the ionotropic gelation process following the report of Jiang et al [31] and slightly optimized, it can ensure the reproducibility of formation in vitro.

P6-MCMs

P6-CMs

3. Are there any positive controls that the authors have used to see the efficiency of the immune response using the modified matrix?

Answers: The experimental components of this experiment are 5 groups, PBS, MCMs, P6, P6-CMs, P6-MCMs. Among them, the MCMs group can serve as positive controls.

4. In Figure 7 experiment, how do the authors differentiate loss of integrity because of active infection and primed defense response by immune cells? If the authors use heat killed or dead bacteria, do they see similar levels of immune cell infiltration in the mice? How does the normal infected lung look??

Answers: We really did not compare the levels of immune cell infiltration in the mice between the aggressive bacteria and dead bacteria. Heat killed or dead bacteria does not have the ability to actively attack, and it is probably different from the extent of damage to tissues caused by aggressive bacteria. It can be clearly seen that the histopathological improvement of mice immunized with microspheres, the lung tissues have obvious bleeding, inflammatory cell infiltration, and tissue texture destruction in normal group.
