# Supplementary material for: Mucosal immunity of mannose-modified chitosan microspheres loaded with the nontyepable Haemophilus influenzae outer membrane protein P6 in BALB/c mice
Source: PLoS One. 2022 Jun 10;17(6):e0269153. doi: 10.1371/journal.pone.0269153 (PMC9187061; doi:10.1371/journal.pone.0269153)
Supplement: Attachment — Submitted filename: Response to Reviewers.docx [file pone.0269153.s004.docx]

Reviewer #2:

1. In response to the comments from the previous revision regarding pathological scoring, authors mentioned that there is a clear visual difference between the groups and hence scoring is not performed. I would like the authors to do scoring done by an independent pathologist to avoid Experiment bias and make a graph to show the difference.?

Answers: Differences between groups were analyzed by pathology scores[32]. To score lung inflammation and damage, the following parameters: edema, interstitial inflamamation, intra-alveolar inflammation, endothelialitis, hyperemia, degree of inflammatory cell infiltration. Each parameter was graded from 0 (absent) to 4 (severe). Nasal mucosa were scored according to the following parameters: presenc and degree of inflammation cell infiltration, presence and degree of the cilia of the nasal mucosa disappearing, degree of looseness of the columnar epithelial cells arranging. Each parameter was graded from 0 (absent) to 3 (severe). The total pathology scores were expressed as the sum of the score for all parametrs.
