# Supplementary material for: Mucosal immunity of mannose-modified chitosan microspheres loaded with the nontyepable Haemophilus influenzae outer membrane protein P6 in BALB/c mice
Source: PLoS One. 2022 Jun 10;17(6):e0269153. doi: 10.1371/journal.pone.0269153 (PMC9187061; doi:10.1371/journal.pone.0269153)
Supplement: Attachment — Submitted filename: Response to Reviewers.docx [file pone.0269153.s005.docx]

Reviewer #3:

1. The authors argue that the observed immune response is because of the enhanced uptake of the microspheres by the macrophages through the mannose receptor mediated endocytosis. While the previous study (Zhu L, et al; ref# 43) suggests that this is a possibility, it is pivotal to show that P6-loaded chitosan upon modification behave as expected. The authors should check the invitro uptake of the microspheres by the receptor expressing cells and use a competition assay to show that it is specific to the receptors. All the immunological parameters show similar degree of differences between P6-CM and P6-MCM (except for the marginal difference in the antibody levels), suggesting that modification did not provide much benefit. So, it is essential to know that the modification is providing additional levels of protection to justify the study.?

Answers:

In this experiment, mannose-modified chitosan microsphers are targeted to the mannose receptor of APC to enhance the humoral and cellular immune effects of the vaccine. The Ig /cytokines experimental datas and literature support (ref#19, 20, 43, 25-27) are sufficient to demonstrate that the mannose-modified chitosan microspheres can target MR in vivo although the direct experimental evidence cannot be supported. In detail: P6-MCMs and P6-CMs exhibited significant statistical differences except on humoral immunity IgA and IgG(Fig. 4). With the cellular immunity , P6-MCMs can trigger higher level of cytokines and CD4+/ CD8+ than P6-CMs(Fig. 5;6), The microsphere vaccines modified with mannose not only enhance cellular immunity but also trigger a mixed Th1/Th2-type immune response, while chitosan microsphere vaccines induce a Th2-type immune response. All these suggest that P6-MCMs enhance immune presentation by targeting APC mannose receptors, thereby enhancing immune responses. Most directly, the previous study (Jiang HL. ref#19 and Cui Z. ref#20) suggest that the mannose-modified chitosan microspheres really target the mannose receptor of RAW264.7 murine macrophage cells。
